# Effects of Aerobic Exercise on Depressive Symptoms in People with Parkinson’s Disease: A Systematic Review and Meta-Analysis of Randomized Controlled Trials

**DOI:** 10.3390/brainsci15080792

**Published:** 2025-07-25

**Authors:** Hao Ren, Yilun Zhou, Yuanyuan Lv, Xiaojie Liu, Lingxiao He, Laikang Yu

**Affiliations:** 1 Beijing Key Laboratory of Sports Performance and Skill Assessment, Beijing Sport University, Beijing 100084, China; renhao11112023@126.com (H.R.); sunflowerlyy@bsu.edu.cn (Y.L.); 2 Department of Strength and Conditioning Assessment and Monitoring, Beijing Sport University, Beijing 100084, China; z987486898@163.com; 3 China Institute of Sport and Health Science, Beijing Sport University, Beijing 100084, China; 4 Department of Pharmacology and Toxicology, Medical College of Wisconsin, Milwaukee, WI 53226, USA; xiaojieliu@mcw.edu; 5 School of Public Health, Xiamen University, Xiamen 361005, China

**Keywords:** aerobic exercise, Parkinson’s disease, depressive symptoms, exercise prescription

## Abstract

**Objectives**: The objective of this study was to assess the effect of aerobic exercise on depressive symptoms and to determine the optimal exercise prescription for Parkinson’s disease (PD) patients. **Methods**: A comprehensive search was conducted across PubMed, Web of Science, Cochrane, Scopus, and Embase databases. A meta-analysis was conducted to determine the standardized mean difference (SMD) and 95% confidence interval. **Results**: Aerobic exercise significantly alleviated depressive symptoms in PD patients (SMD, −0.68, *p* = 0.002). Subgroup analyses revealed that moderate intensity aerobic exercise (SMD, −0.72, *p* = 0.0006), interventions conducted for ≥12 weeks (SMD, −0.85, *p* = 0.04), ≥3 times per week (SMD, −0.68, *p* = 0.002), ≥60 min per session (SMD, −0.57, *p* < 0.0001), and ≥180 min per week (SMD, −0.87, *p* = 0.0002) were more effective in improving depressive symptoms in PD patients, especially in PD patients with a disease duration of ≤6 years (SMD, −1.00, *p* = 0.04). **Conclusions**: Integrating the available data, it is clear that aerobic exercise is a proven method for alleviating depressive symptoms in PD patients. This meta-analysis provides empirical support for clinicians to recommend that PD patients engage in aerobic exercise regimens of no less than 12 weeks’ duration, performed at a minimum frequency of three sessions per week, with each session lasting in excess of 60 min and a cumulative weekly duration of at least 180 min, to effectively attenuate depressive symptomatology. Earlier implementation of aerobic exercise interventions is recommended, as PD patients in the early stages of the disease (up to 6 years post-diagnosis) may derive the greatest benefit in terms of depression symptom improvement from such programs.

## 1. Introduction

Parkinson’s disease (PD), characterized by the degeneration of dopaminergic neurons in the substantia nigra compacta [1,2], manifests clinically through resting tremor, bradykinesia, postural instability, and rigidity [3]. In addition to these motor deficits, PD patients frequently exhibit a broad spectrum of non-motor symptoms, including emotional dysfunction [4], with depressive disorders affecting 2.7% to 90% of patients and major depression impacting up to 17% [5,6]. Historically, depressive symptoms in PD were regarded as secondary to neurodegenerative changes and emerging after motor symptom onset [7]. Nevertheless, prior research has demonstrated a robust correlation between antidepressant use and PD incidence over a two-year period [8]. Furthermore, depression may be implicated in PD’s pathogenesis as a potential precursor to motor symptom development [9,10,11]. This bidirectional interplay between PD and depressive symptoms poses challenges for the clinical diagnosis of depression in PD patients [10,12]. Given that depression can lead to increased suicidal ideation in PD patients [13], it is crucial to prioritize the identification and management of depressive symptoms in this population.

Antidepressant medications and cognitive therapy are currently widely utilized for treating depressive symptoms in PD patients [14,15]. Despite the notable efficacy of these medications in clinical trials, significant depressive symptoms often persist [16]. Moreover, the use of various depression assessment scales in controlled trials has yielded inconsistent results, with many studies expressing dissatisfaction with the available scales [17,18]. Exercise is emerging as a promising adjunctive therapy for alleviating depression, particularly aerobic exercise [19,20]. Research suggests that depressive symptoms in PD patients are associated with alterations in neurotransmitter systems, including dopamine (DA), norepinephrine, and serotonin [19,21]. Aerobic exercise may alleviate depressive symptoms by enhancing the function of these neurotransmitter systems. Animal studies have shown that aerobic exercise can increase D2 receptor mRNA in the rat caudate shell nucleus, thereby boosting DA synthesis [22], and also elevate serotonin levels in rats [23]. Clinical evidence from a randomized controlled trial (RCT) indicates that 12 weeks of aerobic exercise is more effective than physiotherapy in reducing depressive symptoms in PD patients [24].

However, a recent meta-analysis, which included only five studies on aerobic exercise interventions and identified substantial publication bias, suggested that aerobic exercise may not significantly affect depressive symptoms in PD patients [25]. Several meta-analyses have examined the effects of exercise on depressive symptoms in PD patients. Although these studies generally support a positive association between exercise and symptom improvement, they offer little practical guidance for clinicians. Critical details such as optimal exercise intensity, session duration, and frequency remain unclear. To address these gaps, we conducted a comprehensive systematic review and meta-analysis of RCTs to evaluate the effects of aerobic exercise on depressive symptoms in PD patients and to determine the optimal exercise prescription for this population.

## 2. Materials and Methods

This meta-analysis was executed in accordance with the Preferred Reporting Items for Systematic Reviews and Meta-Analysis (PRISMA) guidelines [26]. The protocol has been registered on PROSPERO (CRD42023459353).

### 2.1. Search Strategy

An extensive search was executed across five databases: PubMed, Web of Science, Cochrane, Scopus, and Embase, from inception to 11 April 2024. Search terms included keywords and MeSH terms: exercise, Parkinson’s disease, and depression. Two authors (H.R. and Y.Z.) independently carried out the search. In cases of disagreement between the two authors, a third author (L.Y.) would be consulted to reach a consensus through discussion.

### 2.2. Eligibility Criteria

Studies meeting the following criteria were included: (1) RCTs; (2) involving both an aerobic exercise intervention group and a control group; (3) employing depression scales as the outcome measure. Publications not in English, animal model studies, reviews, and conference papers were excluded.

### 2.3. Data Extraction

Two authors (H.R. and Y.Z.) independently conducted data extraction, which mainly included the following: (1) study features (last name of the first author, publication year); (2) intervention features (duration, intensity, frequency, session length); (3) participant features (sample size, gender, age, disease duration); and (4) intervention effects (changes in depression from baseline to post-intervention).

### 2.4. Methodological Quality Assessment

Two authors (H.R. and Y.Z.) appraised the methodological quality of the included studies using the Cochrane risk of bias criteria, which consist of seven items [27,28]. Each item was classified as “low risk”, “unclear risk”, or “high risk” based on responses to the signaling questions. Two authors independently conducted the methodological quality assessment. When disagreements occurred between the two authors, a third author (L.Y.) joined the discussion until consensus was achieved.

### 2.5. Statistical Analysis

Mean and standard deviation (SD) values reflecting the change from baseline to post-intervention were extracted from each study to summarize the effect. For studies reporting standard errors (SE) or 95% confidence intervals (CI), SD was calculated using the formula previously described [29].

Because depressive symptoms were measured with different questionnaires, we used a random-effect model to pool the data and calculated the standardized mean difference (SMD) with 95% CI. Heterogeneity was assessed using the I^2^ statistic. I^2^ < 25% indicates no significant heterogeneity, 25% < I^2^ < 50% indicates low heterogeneity, 50% < I^2^ < 75% indicates moderate heterogeneity, and I^2^ > 75% indicates high heterogeneity. In cases of high heterogeneity, subgroup analysis and sensitivity analysis were employed to interpret the results. For the overall effect, *p* < 0.05 was considered statistically significant.

## 3. Results

### 3.1. Study Selection

**Figure 1:**
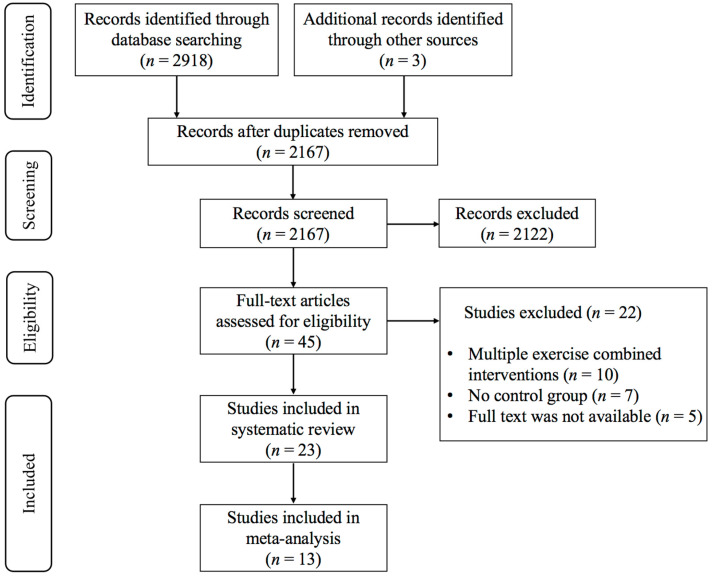
PRISMA flowchart of study selection.

As shown in Figure 1, the initial search yielded a total of 2918 studies, and 3 studies were identified through other sources. After eliminating duplicates, 2167 studies remained, of which 2122 were ineligible for inclusion based on title and abstract screening. Twenty-two studies were excluded by reading the full text of 45 studies: (1) multiple-exercise combined interventions (n = 10), (2) there was no control group (n = 7), and (3) the full text was not available (n = 5). Finally, 23 studies [30,31,32,33,34,35,36,37,38,39,40,41,42,43,44,45,46,47,48,49,50,51,52] met the inclusion criteria.

### 3.2. Characteristics of the Included Studies

The main characteristics of the participants and the interventions are shown in Table S1. There were 288 and 267 participants in the aerobic exercise and control groups, respectively. All studies reported aerobic interventions, including cycling [40,51,52], dancing [43,46,47,49], walking [41], yoga [38,44], Qigong [37,45,48], arm crank [31], body awareness training [34,42], boxing [35,39], and multimodal physical exercise [32,33,36,50]. The overall duration ranged from 5 to 24 weeks, with a mean intervention duration of 12.08 weeks. The frequency ranged from 1 to 5 times, with a mean frequency of 2.34 times per week. The session duration ranged from 20 to 90 min, with a mean session duration of 46.66 min. The weekly time was at least 60 min per week, with a mean weekly time of 160 min per week. Depressive symptoms were assessed using several outcome measures, including the Beck Depression Inventory (BDI) [30,31,34,36,39,40,42,45,46,47,51], Beck Depression Inventory-II (BDI-II) [33,41,49], Self-Rating Depression Scale (SDS) [43], Hospital Anxiety and Depression Scale (HADS-depression) [32,44,50,52], Geriatric Depression Scale (GDS) [38,48], and Epidemiologic Studies Depression (CES-D) [35].

In line with a previous study [53], we categorized the intensity of aerobic exercise as follows: 20% < maximal oxygen uptake (VO_2_max) < 40%, 40% < maximal heart rate (HRmax) < 55%, 20% < heart rate reserve (HRR) < 40%, or 8 < rating of perceived exertion (RPE) < 10 were determined as low-intensity; 40% < VO_2_max < 60%, 55% < HRmax < 70%, 40% < HRR < 60%, or 11 < RPE < 13 were determined as moderate-intensity; 60% < VO_2_max < 85%, 70% < HRmax < 90%, 60% < HRR < 85%, or 14 < RPE < 16 were determined as vigorous-intensity. Among the 13 included studies, 7 studies [42,44,45,46,47,48,49] did not describe the intensity of exercise, 3 studies [41,50,52] performed moderate-intensity aerobic exercise, and 3 studies [41,50,52] performed vigorous-intensity aerobic exercise.

### 3.3. Meta-Analysis Results

**Figure 2:**
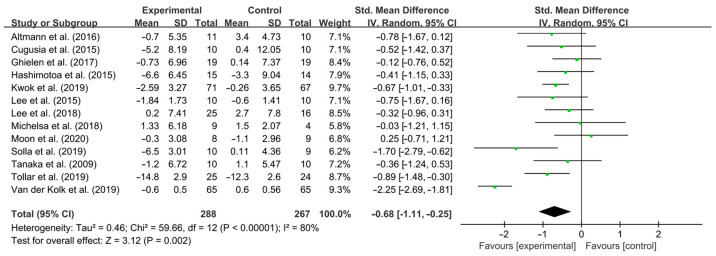
Meta-analysis results of the effect of aerobic exercise on depressive symptoms in PD patients [40,41,42,43,44,45,46,47,48,49,50,51,52].

Aerobic exercise had a significant effect on improving depressive symptoms in PD patients [SMD, −0.68 (95% CI, −1.11 to 0.25), *p* = 0.002, I^2^ = 80% (high heterogeneity), Figure 2]. To explain the heterogeneity between included studies and find modifiable factors of aerobic exercise, subgroup analysis and sensitivity analysis were further performed.

### 3.4. Subgroup Analysis

**Figure 3:**
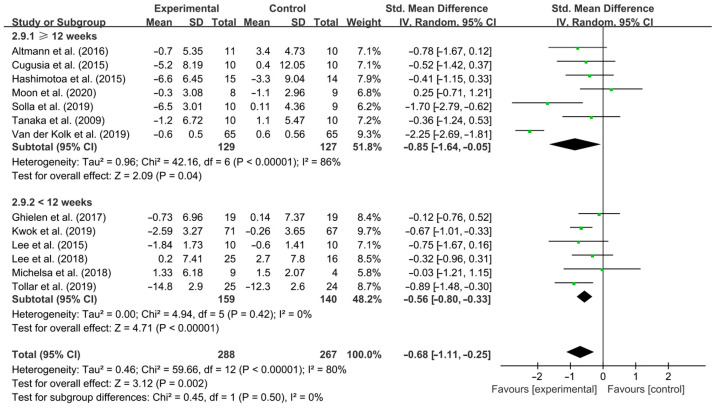
Meta-analysis results of the effect of aerobic exercise intervention on depressive symptoms in PD patients [40,41,42,43,44,45,46,47,48,49,50,51,52].

Stratifying the analysis by intervention duration, <12 weeks [SMD, −0.56 (95% CI, −0.80 to −0.33), *p* < 0.00001, I^2^ = 0% (no heterogeneity)] and ≥12 weeks [SMD, −0.85 (95% CI, −1.64 to −0.05), *p* = 0.04, I^2^ = 86% (high heterogeneity), Figure 3] of aerobic exercise significantly improved depressive symptoms in PD patients, with ≥12 weeks of aerobic exercise having a greater effect.

**Figure 4:**
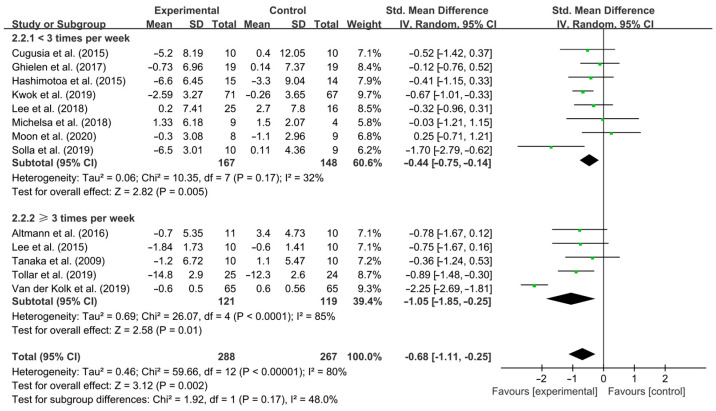
Meta-analysis results of the effect of frequency of aerobic exercise on depressive symptoms in PD patients [40,41,42,43,44,45,46,47,48,49,50,51,52].

In addition, aerobic exercise conducted <3 times per week [SMD, −0.44 (95% CI, −0.75 to −0.14), *p* = 0.005, I^2^ = 32% (low heterogeneity)] and ≥3 times per week [SMD, −1.05 (95% CI, −1.85 to −0.25), *p* = 0.01, I^2^ = 85% (high heterogeneity), Figure 4] significantly improved depressive symptoms in PD patients, with aerobic exercise conducted ≥3 times per week having a greater effect.

**Figure 5:**
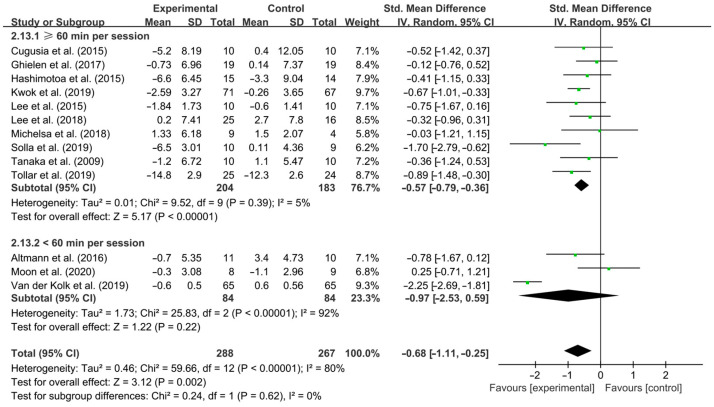
Meta-analysis of the effect of duration of aerobic exercise per session on depressive symptoms in PD patients [40,41,42,43,44,45,46,47,48,49,50,51,52].

Aerobic exercise conducted for ≥60 min per session significantly improved depressive symptoms in PD patients [SMD, −0.57 (95% CI, −0.79 to −0.36), *p* < 0.0001, I^2^ = 5% (low heterogeneity)], while aerobic exercise conducted for < 60 min per session had no significant effect on improving depressive symptoms in PD patients [SMD, −0.97 (95% CI, −2.53 to 0.59), *p* = 0.22, I^2^ = 92% (high heterogeneity), Figure 5].

**Figure 6:**
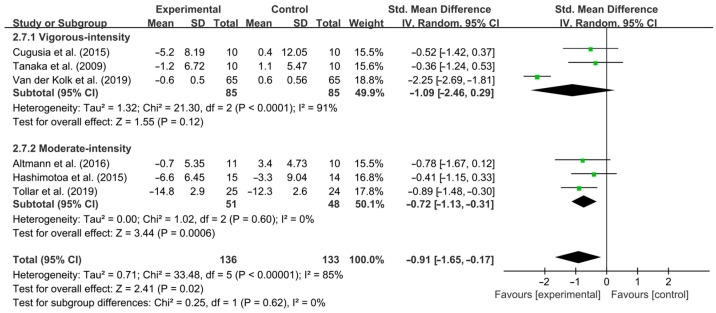
Meta-analysis results of the effect of different intensities of aerobic exercise on depressive symptoms in PD patients [40,41,43,50,51,52].

Furthermore, moderate-intensity aerobic exercise significantly improved depressive symptoms [SMD, −0.72 (95% CI, −1.13 to −0.31), *p* = 0.0006, I^2^ = 0% (no heterogeneity)], while vigorous-intensity aerobic exercise had no significant effect on improving depressive symptoms in PD patients [SMD, −1.09 (95% CI, −2.46 to 0.29), *p* = 0.12, I^2^ = 91% (high heterogeneity), Figure 6].

**Figure 7:**
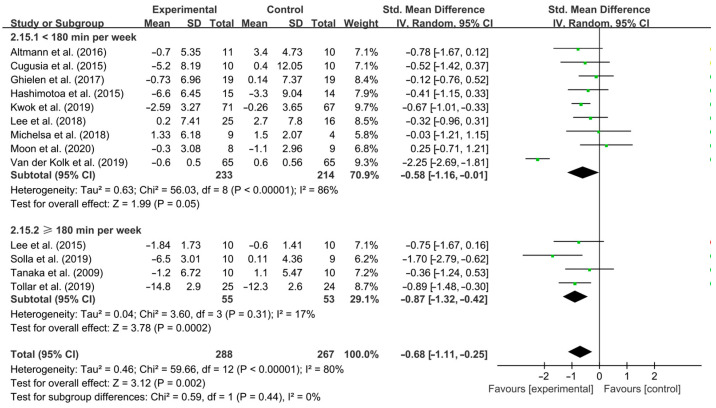
Meta-analysis of the effect of duration of aerobic exercise per week on depressive symptoms in PD patients [40,41,42,43,44,45,46,47,48,49,50,51,52].

Moreover, aerobic exercise conducted for <180 min per week [SMD, −0.58 (95% CI, −1.16 to −0.01), *p* = 0.05, I^2^ = 86%] and ≥180 min per week [SMD, −0.87 (95% CI, −1.32 to −0.42), *p* = 0.0002, I^2^ = 17%, Figure 7] significantly improved depressive symptoms in PD patients, with aerobic exercise conducted for ≥180 min per week having a greater effect.

**Figure 8:**
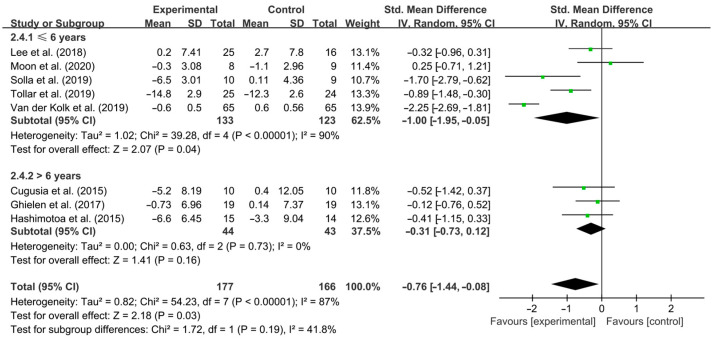
Meta-analysis of the effect of aerobic exercise on depressive symptoms in PD patients with different disease durations [41,42,43,45,48,49,50,51,52].

Finally, aerobic exercise significantly improved depressive symptoms in PD patients with a disease duration of ≤6 years [SMD, −1.00 (95% CI, −1.95 to −0.05), *p* = 0.04, I^2^ = 90% (high heterogeneity)], while aerobic exercise had no significant effect on improving depressive symptoms in PD patients with a disease duration of >6 years [SMD, −0.31 (95% CI, −0.73 to 0.12), *p* = 0.16, I^2^ = 0% (no heterogeneity), Figure 8].

### 3.5. Risk of Bias

The methodological quality of the included studies was assessed using the Cochrane risk assessment tool, focusing on six key aspects: selection bias, performance bias, detection bias, attrition bias, reporting bias, and other potential biases [54]. As depicted in Figure S1, among the included studies, one study (7.7%) provided low-quality evidence, three studies (23.1%) offered moderate-quality evidence, and nine studies (69.2) presented high-quality evidence.

### 3.6. Publication Bias

We assessed potential publication bias by generating a funnel plot (Figure S2). Although visual inspection suggested some asymmetry, Egger’s test indicated that small-study effects did not significantly influence the results for depressive symptoms (*p* = 0.071, Table S2).

### 3.7. Sensitivity Analysis

The sensitivity analysis demonstrated that the overall effect of aerobic exercise on alleviating depressive symptoms in PD patients remained stable. Specifically, the direction and magnitude of the effect were consistent, even when any single study was excluded from the analysis (Figure S3).

## 4. Discussion

### 4.1. Main Findings

Our findings indicated that aerobic exercise significantly alleviated depressive symptoms in PD patients, which aligns with the previous studies [25,55,56,57]. Subgroup analyses revealed that moderate-intensity aerobic exercise, interventions lasting ≥12 weeks, ≥3 times per week, and ≥60 min per session were more effective in alleviating depressive symptoms, particularly in patients with a disease duration of ≤6 years.

### 4.2. Effects of Aerobic Exercise on Depressive Symptoms in PD Patients

Aerobic exercise improves depressive symptoms in PD patients through several potential mechanisms. Firstly, aerobic exercise alleviates depression in PD patients by improving neurotransmitter dysregulation [58]. It is universally acknowledged that the pathological hallmark of PD is the accumulation of filamentous cytoplasm primarily composed of α-synuclein aggregates [59], which further induces neuronal degeneration and even death [60,61]. Dysfunction of central dopaminergic neurotransmission is associated with depression. When pathological features extend to DA, dysregulation of DA release, coupled with alterations in the expression or function of DA receptors, can exacerbate depressive symptoms in PD patients, potentially resulting in dysregulation of neural circuits involved in mood regulation and reward mechanisms [62,63]. Concurrently, in the pharmacological treatment of depressive symptoms in PD, the dopaminergic system is frequently targeted for therapy [64]. It has been demonstrated that following prolonged aerobic exercise intervention, PD patients can release more DA, and an increase in DA receptor availability, accompanied by activation of the ventral striatum, has been observed [65,66]. This may account for one of the mechanisms by which aerobic exercise improves depressive symptoms in PD patients. Apart from the dopaminergic system, neurotransmitters such as the norepinephrine (NE) and serotonin (5-HT) systems independently impact and interact with depressive symptoms in PD patients [19,58,67]. The locus coeruleus (LC) serves as the primary source of NE, and α-synuclein aggregation initially occurs in the LC, subsequently causing degeneration of the LC. This leads to reduced NE levels and increased depressive symptoms in PD patients [19,68,69].

Studies suggest that 5-HT damage at cortical margins is correlated with the severity of depressive symptoms in PD patients, and neurodegeneration of the serotonergic system is observed to be more pronounced in PD patients with depressive symptoms compared to those without depressive symptoms [70,71]. Additionally, 5-HT serves as a target for pharmacological therapy in the treatment of depressive symptoms, and selective serotonin reuptake inhibitors exhibit more potent antidepressant effects in PD patients [19,72]. Aerobic exercise has been shown to reduce depressive symptoms by modulating 5-HT receptors in rats [23,73]. An RCT demonstrated that aerobic exercise can alleviate depressive symptoms in PD patients by enhancing the serotonergic system [74] and modulating non-DA signals related to depressive symptoms [19,75].

Secondly, aerobic exercise improves depressive symptoms in PD patients by modulating neuroinflammation [76]. It has also been proposed that inflammation may not be an absolute prerequisite for depressive symptoms [77], but rather, the extent of microglia damage and microRNA dysregulation due to neuroinflammation may be more closely tied to depressive symptoms [78,79]. In a treadmill exercise experiment on aged rats, aerobic exercise was found to reduce neuroinflammatory symptoms [80], while Sliva et al. [81] demonstrated that prolonged aerobic exercise decreases the expression of markers associated with neuroinflammation and glucocorticoid mRNA receptors. It has been shown that aerobic exercise can mitigate microglia-mediated neuroinflammation by inhibiting NLRP3 expression in microglia [82]. Furthermore, aerobic exercise can be utilized to alleviate depressive symptoms in PD patients by decreasing inflammatory markers, such as interleukin, homocysteine, and tumour necrosis factor-α (TNF-α) [80,83].

Thirdly, aerobic exercise alleviates depressive symptoms in PD patients by improving dysregulation of neurotrophic factors [84]. Previous studies have demonstrated that reduced serum brain-derived neurotrophic factor (BDNF) levels may signify the presence of depressive symptoms in PD patients. Lower serum BDNF levels were observed in depressed PD patients compared to non-depressed PD patients, and these levels were negatively correlated with the severity of depressive symptoms in PD patients [85,86]. Knaepe et al. [87] showed that aerobic exercise significantly increased serum BDNF levels, which is in agreement with the findings of Mackay et al. [88]. Our previous study also found that aerobic exercise elevated hippocampal and cortical BDNF levels in patients with progressive neurodegenerative diseases, such as Alzheimer’s disease (AD) [89]. Another meta-analysis has shown that abnormal levels of BDNF are likely to be implicated in pathological processes in PD patients, regardless of the presence or absence of depressive symptoms [90]. Therefore, aerobic exercise modifies the vasculature system in the brain, elevates the levels of BDNF, modulates the neurochemistry and neuroplasticity of the brain, and promotes neurogenesis [91,92], ultimately leading to the alleviation of depressive symptoms in PD patients.

Finally, two of the included studies [42,44] reported follow-up results, 18 and 20 weeks post-intervention cessation, respectively, demonstrating varying degrees of depressive symptoms in the intervention groups. This may suggest that the benefits of aerobic exercise must be maintained to persist. A recent meta-analysis reported that aerobic exercise did not alleviate depressive symptoms in PD patients [25], a conclusion that conflicts with our findings. We believe this discrepancy may arise for two reasons: first, that analysis included only a limited number of aerobic-exercise trials, increasing the risk of publication bias; second, the results of large-sample studies within its subgroups may have overshadowed those of smaller trials, warranting cautious interpretation.

### 4.3. Effects of Different Exercise Design Parameters on Depressive Symptoms in PD Patients

Our subgroup analysis showed that <12 weeks and ≥12 weeks of aerobic exercise significantly improved depressive symptoms in PD patients, with ≥12 weeks of aerobic exercise having a greater effect, which is consistent with a previous study [93]. A meta-analysis examining aerobic exercise in depressed individuals also found that a longer duration of aerobic exercise intervention led to a greater reduction in depressive symptoms [94]. Likewise, an RCT demonstrated remission of depressive symptoms after 6 weeks of moderate-intensity aerobic exercise in depressed patients, with some patients exhibiting a sustained decrease in depression scores during the follow-up phase [95], perhaps due to the fact that the beneficial effects of aerobic exercise on depressive symptoms take time to manifest fully. Enhanced serotonergic system function may underlie this phenomenon, as Melancon et al. [74] observed that sustained increases in brain serotonergic activity facilitated more potent antidepressant effects in older adults engaging in over 12 weeks of aerobic exercise. In addition, Mackay et al. [88] suggested that >12 weeks of aerobic exercise is an optimal duration for promoting elevated serum BDNF levels. Furthermore, prolonged aerobic exercise improves aerobic fitness levels in PD patients, which is associated with elevated non-displaceable binding potential in the striatum [96], thereby yielding a more prolonged ameliorative effect on the DA system and subsequently diminishing depressive symptoms in PD patients. Therefore, the antidepressant effect of aerobic exercise in PD patients may exhibit a cumulative pattern, with longer interventions resulting in superior improvements in neurotransmitters and neurotrophic factors, and fostering functional changes in brain structure [93].

In terms of the intervention frequency, we adopted the American College of Sports Medicine’s (ACSM) recommendation of three times per week of aerobic exercise for PD patients [57] as a benchmark to investigate the optimal frequency. Our results showed that aerobic exercise conducted <3 times per week and ≥3 times per week significantly improved depressive symptoms in PD patients, with aerobic exercise conducted ≥3 times per week having a greater effect. A meta-analysis indicated that the frequency of exercise interventions significantly influenced serum BDNF levels [88]. Additionally, another study showed that aerobic exercise conducted ≥3 times per week significantly increased serum BDNF levels, whereas aerobic exercise <3 times per week had no significant effect [87]. Considering the link between frequency and neuroinflammation, an animal study showed that aerobic exercise conducted four times per week could mitigate neuroinflammation in the brain [97]. Therefore, we hypothesized that engaging in aerobic exercise ≥ 3 times per week could modulate neurotrophic factors in the brain, reduce inflammation, and consequently alleviate depressive symptoms in PD patients.

In addition, the frequency of aerobic exercise interventions may correlate with modifications in brain structure, as evidenced by a recent study revealing that PD patients engaging in aerobic exercise three times per week exhibited reduced brain atrophy [98]. However, it is crucial to emphasize that the frequency of aerobic exercise is not the sole determinant in alleviating depressive symptoms in PD patients, as factors such as session duration also play a significant role. Therefore, we cannot prescribe a precise intervention frequency, and the current evidence is not sufficient to endorse an optimal combination of factors.

Our subgroup analysis indicated that aerobic exercise conducted for ≥60 min per session significantly improved depressive symptoms, while aerobic exercise conducted for <60 min per session had no significant effect on improving depressive symptoms in PD patients. While some studies have suggested that 45–60 min of aerobic exercise can reduce depressive symptoms [99], it is acknowledged that high-quality aerobic exercise can be challenging for PD patients, particularly those with pronounced tremor and mobility issues. Consequently, a suitable extension of exercise duration is vital to ensure its efficacy. Furthermore, given that PD patients may grapple with diminished self-esteem, which exacerbates depressive symptoms, the social support and moral encouragement received during aerobic exercise may contribute to alleviating their depressive symptoms [100]. By completing longer aerobic exercises, PD patients can foster a stronger sense of self-confidence. It is worth noting that engaging in exercise for excessive durations can also result in adverse health effects [101]. Consequently, it is imperative to consider the combined influence of factors like the intensity of the intervention alongside the session duration.

To examine the joint influence of frequency and session duration, we performed a subgroup analysis anchored to the guideline of three weekly sessions (≥60 min per session), yielding a cumulative weekly volume of 180 min. Interventions accumulating both <180 min and ≥180 min per week significantly alleviated depressive symptoms in PD patients. However, the ≥180 min regimen elicited a markedly larger effect. This pattern indicates a dose–response relationship between aerobic exercise and the alleviation of depressive symptoms in PD. Weekly volumes reaching or exceeding 180 min appear to provide sufficient stimulus to activate neuroprotective cascades, including hippocampal volumetric expansion, elevated BDNF [87,89,91], suppression of chronic neuroinflammation and oxidative stress [82], and pronounced dopaminergic modulation [65,66], ultimately culminating in diminished depressive symptomatology in PD.

It is a widely accepted fact that moderate- and vigorous-intensity aerobic exercises are superior to low-intensity aerobic exercise in alleviating depressive symptoms in PD patients. However, fewer studies have delved into the specific differences between the effects of moderate- and vigorous-intensity aerobic exercise. To address this gap, we conducted a subgroup analysis based on the intensity. Our findings showed that moderate-intensity aerobic exercise significantly improved depressive symptoms, while vigorous-intensity aerobic exercise had no significant effect on improving depressive symptoms in PD patients, which is consistent with a previous study [55]. One possible explanation is that moderate-intensity aerobic exercise promotes elevated serum BDNF levels, whereas the promoting effect of vigorous-intensity aerobic exercise is less pronounced [87], a phenomenon that is more evident in populations with chronic diseases [102]. This may stem from a dose–response relationship between intensity and BDNF levels, where individuals with chronic diseases tend to benefit more from moderate-intensity aerobic exercise [87]. In addition, prolonged moderate-intensity aerobic exercise triggers an increase in DA release and receptor numbers. In an animal study, it was observed that moderate-intensity treadmill exercise induced an upregulation of D2 receptors [22]. Furthermore, in a rat model of PD, a four-week moderate-intensity treadmill exercise was sufficient to modulate striatal DA and glutamate signaling, thereby enhancing the dopaminergic system [103]. Moreover, it is now widely acknowledged that PD is more prevalent among the elderly population [28], for whom performing vigorous-intensity aerobic exercise poses greater challenges. A meta-analysis has indicated a higher incidence of severity in vigorous-intensity aerobic exercise compared to moderate-intensity aerobic exercise [104], emphasizing that the injuries sustained during vigorous-intensity aerobic exercise can disrupt training continuity, ultimately impacting the overall outcome.

Ultimately, it is crucial to recognize that PD may or may not exhibit alterations in response to aerobic exercise as the duration of disease onset increases. To gain a deeper understanding of this phenomenon, we conducted a subgroup analysis based on participants’ disease duration. Our results showed that aerobic exercise significantly improved depressive symptoms in PD patients with a disease duration of ≤6 years, while aerobic exercise had no significant effect on improving depressive symptoms in PD patients with a disease duration of >6 years. This may stem from the fact that aerobic exercise, by enhancing physiological indices, can induce structural brain changes in PD patients [93], thereby delaying depressive symptoms associated with the disease process. However, as PD progresses, aerobic exercise becomes less effective as an intervention, particularly among those who did not initiate aerobic exercise interventions at an earlier stage. Given that depressive symptoms can manifest early in the course of PD [1], we therefore advise PD patients to commence aerobic exercise interventions as early as possible.

### 4.4. Limitations

This study had certain limitations. Firstly, the included studies were RCTs focusing on aerobic exercise interventions, which inherently could not be fully blinded. Therefore, subjective factors may introduce a degree of bias into the quality assessment. In addition, there is a high degree of heterogeneity among the studies, which might stem from multiple risk factors, including complications of PD and the adverse impact of poor lifestyle habits. Furthermore, prior research has demonstrated the effectiveness of resistance exercise in alleviating depressive symptoms in PD patients [105]. However, our study solely focused on the effects of aerobic exercise, neglecting the potential benefits of resistance exercise or combined exercise approaches. Finally, given the significant heterogeneity observed in the meta-analysis results, our findings should be interpreted with caution.

## 5. Conclusions

Integrating the available data, it is clear that aerobic exercise is a proven method for alleviating depressive symptoms in PD patients. This meta-analysis provides empirical support for clinicians to recommend that PD patients engage in aerobic exercise regimens of no less than 12 weeks’ duration, performed at a minimum frequency of three sessions per week, with each session lasting in excess of 60 min and a cumulative weekly duration of at least 180 min, to effectively attenuate depressive symptomatology. Earlier implementation of aerobic exercise interventions is recommended, as PD patients in the early stages of the disease (up to 6 years post-diagnosis) may derive the greatest benefit in terms of depression symptom improvement from such programs.

## Data Availability

All data generated or analyzed during this study are included in the article/supplementary material.

## Supplementary Materials

The following supporting information can be downloaded at https://www.mdpi.com/article/10.3390/brainsci15080792/s1, Figure S1: Results of Cochrane risk of bias tool; Figure S2: Funnel plot; Figure S3: Sensitivity analysis results; Table S1: Characteristics of studies included in this meta-analysis; Table S2: Results of Egger’s test.

## Abbreviations

The following abbreviations are used in this manuscript:

PD	Parkinson’s disease
DA	Dopamine
RCT	Randomized controlled trial
SD	Standard deviation
SE	Standard error
CI	Confidence interval
SMD	Standardized mean difference
EG	Experimental group
CG	Control group
NR	No report
BDI	Beck Depression Inventory
BDI-II	Beck Depression Inventory-II
SDS	Self-rating Depression Scale
HADS	Hospital Anxiety and Depression Scale
GDS	Geriatric Depression Scale
CES-D	Epidemiologic Studies Depression
VO_2_max	Maximal oxygen uptake
HRmax	Maximal heart rate
HRR	Heart rate reserve
RPE	Rating of perceived exertion
NE	Norepinephrine
5-HT	Serotonin
LC	Locus coeruleus
RNA	Ribonucleic Acid
NLRP3	NOD-like receptor protein 3
TNF-α	Tumour necrosis factor-α
BDNF	Brain-derived neurotrophic factor
AD	Alzheimer’s disease
ACSM	American College of Sports Medicine’s
