# Supplementary material for: Effects of Aerobic Exercise on Depressive Symptoms in People with Parkinson’s Disease: A Systematic Review and Meta-Analysis of Randomized Controlled Trials
Source: Brain Sci. 2025 Jul 25;15(8):792. doi: 10.3390/brainsci15080792 (PMC12384485; doi:10.3390/brainsci15080792)
Supplement: Supplementary file 1 [file brainsci-15-00792-s001.zip › brainsci-3725614-supplementary.pdf]

Supplementary Material

Effects of aerobic exercise on depressive symptoms in people with Parkinson's disease: a systematic review and meta-analysis of randomized controlled trials

Figure S1. Results of Cochrane risk of bias tool 2
Figure S2. Funnel plot 3
Figure S3. Sensitivity analysis results 4
Table S1. Characteristics of studies included in this meta-analysis 5
Table S2. Results of Egger's test 7

Figure S1. Results of Cochrane risk of bias tool

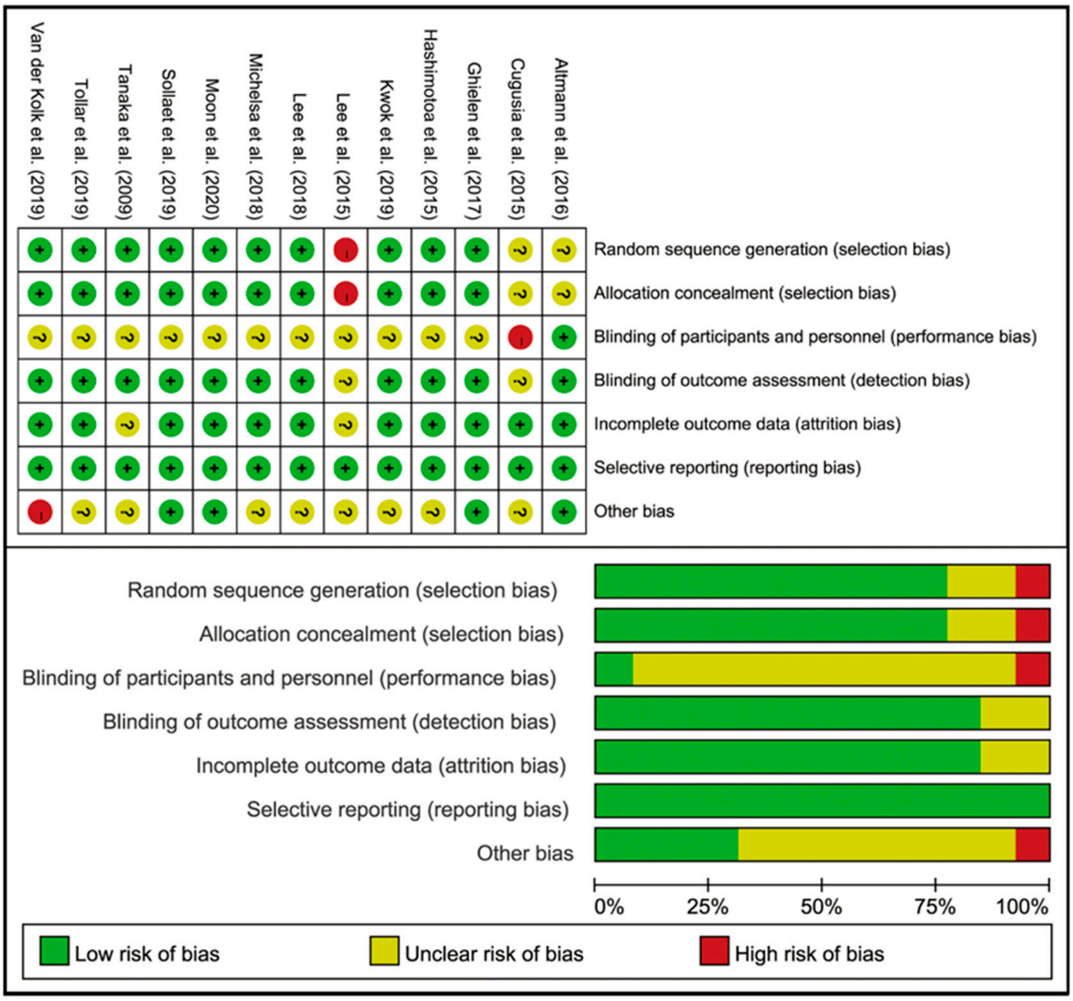

Figure S2. Funnel plot

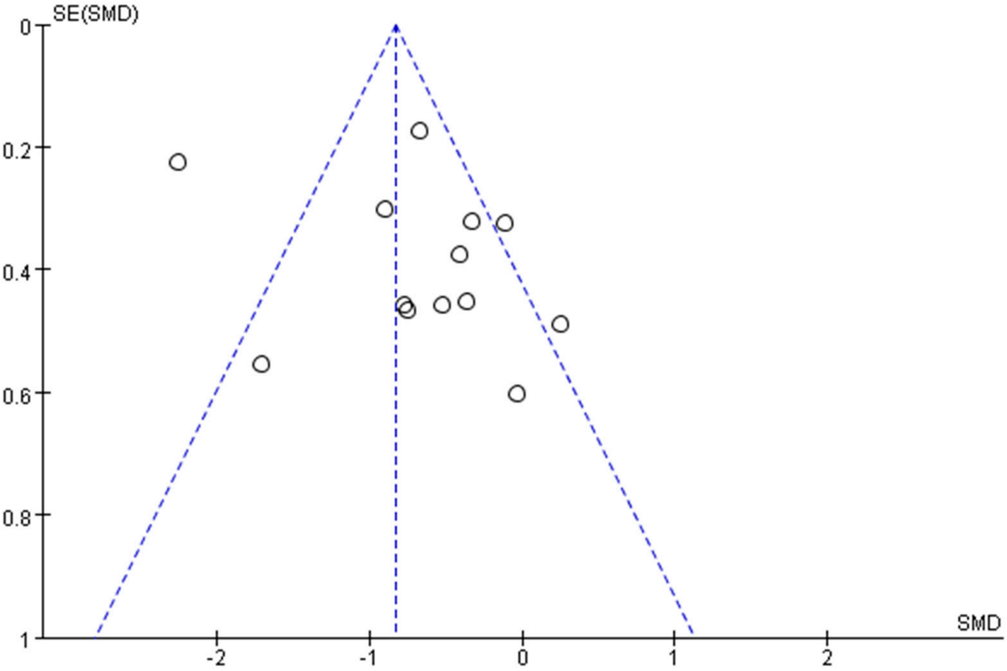

Figure S3. Sensitivity analysis results

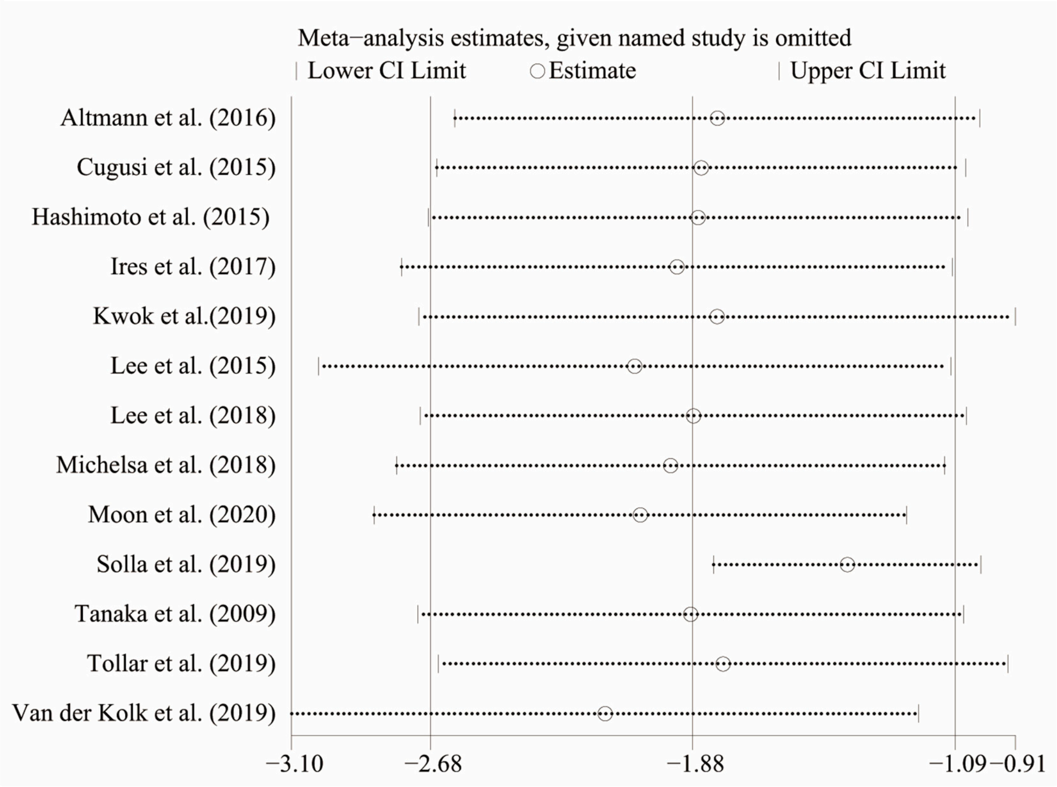

Table S1. Characteristics of studies included in this meta-analysis

Study	Sample size	Age (y)	Stage of disease	Disease duration (y)	Intervention	Characteristics of Interventions	Outcome measures
Altmann et al. (2016)	EG = 11<br>CG = 10	EG: 62.8 (8.6)<br>CG: 67.8 (9.8)	Total: H&Y stage 1-3	NR	EG: Aerobic exercise<br>CG: Normal activities	20-45 min/session, 50-75% HRR, three sessions per week for 16 weeks	BDI
Cugusi et al. (2015)	EG = 10<br>CG = 10	EG: 68.1 (8.7)<br>CG: 66.6 (7.3)	Total: H&Y stage 1-3	EG:7(2)<br>CG:7(4)	EG: Nordic Walking<br>CG: Conventional care	60 min/session, 60-80% HRR, two session per week for 12 weeks	BDI-II
Hashimoto et al. (2015)	EG = 15<br>CG = 14	EG: 67.9 (7.0)<br>CG: 69.7 (4.0)	UPDRS,<br>IG: 42.7 (13.9)<br>CG: 33.4 (14.2)	EG: 6.3 (4.6)<br>CG: 7.8 (6.2)	EG: Dance<br>CG: Normal lives	60 min/session, one session per week for 12 weeks	SDS
Ghielen et al. (2017)	EG = 19<br>CG = 19	EG: 59.6 (9.7)<br>CG: 66.6 (8.4)	Total: H&Y stage 2-3	EG: 10.5 (5.7)<br>CG: 12.3 (4.3)	EG: BEWARE<br>CG: Active control	60 min/session, two session per week for 6 weeks	BDI
Kwok et al. (2019)	EG = 71<br>CG = 67	EG: 63.7 (8.2)<br>CG: 63.5 (9.3)	Total: H&Y stage 1-3	NR	EG: Yoga<br>CG: Active control	EG: 90min/session, one session per week for 8 weeks<br>CG: 60min/session, one session per week for 8 weeks	HADS-depression
Lee et al. (2015)	EG = 10<br>CG = 10	EG: 68.4 (2.9)<br>CG: 70.1 (3.3)	Total: H&Y stage 1-3	NR	EG: Virtual reality dance exercise<br>CG: NDT and FES	EG:75 min/session, five session per week for 6 weeks<br>CG: 45min/session, five session per week for 6 weeks	BDI
Lee et al. (2018)	EG = 25<br>CG = 16	EG: 65.8 (7.2)<br>CG: 65.7 (6.4)	Total: H&Y stage 1-3	EG: 4.5 (3.3)<br>CG: 4.4 (3.0)	EG: QI dance<br>CG: Normal lives	60 min/session, two sessions per week for 8 weeks	BDI

Michels et al. (2018)	EG = 9<br>CG = 4	EG: 66.44<br>CG: 75.50	NR	NR	EG: Dance<br>CG: No actual physical exercises	60 min/session, one session per week for 12 weeks	BDI
Moon et al. (2020)	EG = 8<br>CG = 9	EG: 66.4 (8.1)<br>CG: 65.9 (5.4)	Total: H&Y stage 1-3	EG: 4.25 (2.1)<br>CG: 5.33 (3.3)	EG: Qigong<br>CG: Sham Qigong group	45-60 min/session, one session per week for 12 weeks	GDS
Sollaet al. (2019)	EG = 10<br>CG = 9	EG: 67.8 (5.9)<br>CG: 67.1 (6.3)	Total: H&Y stage 1-3	EG: 4.4 (4.5)<br>CG: 5.0 (2.9)	EG: Sardinian Folk Dance<br>CG: Normal lives	90 min/session, two sessions per week for 12 weeks	BDI-II
Tanaka et al. (2009)	EG = 10<br>CG = 10	EG: 64.8 (8.49)<br>CG: 64.6 (6.25)	H&Y,<br>IG: 1.75 (0.82)<br>CG: 1.40 (0.45)	NR	EG: Aerobic predominance<br>CG: A wait-listed control	60 min/session, 60–80% HRmax, there sessions per week for 24 weeks	HADS-depression
Tollar et al. (2019)	EG = 25<br>CG = 24	EG: 70.6(4.10)<br>CG: 67.5(4.28)	H&Y,<br>IG: 2.4 (0.51)<br>CG: 2.4 (0.51)	EG: 7.5 (2.16)<br>CG: 7.3 (2.21)	EG: Cycling<br>CG: A wait-listed control	60 min/session, 80% HRmax or RPE 12 - 13, five sessions per week for 5 weeks	BDI
Van der Kolk et al. (2019)	EG = 65<br>CG = 65	EG: 59.3(8.3)<br>CG: 59.4(9.3)	Total: H&Y stage 1-2	EG: 3.41<br>CG: 3.12	EG: Aerobic exercise<br>CG: Active control	45 min/session, 50%-80% HRR, three sessions per week for 24 weeks	HADS-depression

Abbreviations: PD, Parkinson’s disease; EG, experimental group; CG, control group; NR, no report; H&Y, Hoehn and Yahr; UPDRS, Unified-Parkinson Disease Rating Scale, NDT, neurodevelopment treatment; FES, functional electrical stimulation; BDI, Beck Depression Inventory; BDI-II, Beck Depression Inventory II; SDS, Self-rating Depression Scale, HADS Hospital Anxiety and Depression Scale; GDS, Geriatric Depression Scale; HRmax, maximum heart rate; HRR, heart rate reserve; PRE, Rating of Perceived Exertion.

Table S2. Results of Egger's test

Std_Eff	Coef.	Std. err.	t	P > |t|	95% CI
slope	-1.14468	0.121821	-9.4	0	-1.4128
bias	-0.75075	0.375713	-2	0.071	-1.57769

Abbreviations: Coef, coefficient; Std. Err, standard error; t, t-test statistic; P, probability; CI, confidence interval.
